# High Voltage, High Stakes: Assessing Cardiovascular Effects of Electromagnetic Fields From Electric Vehicles

**DOI:** 10.1016/j.jacasi.2025.09.012

**Published:** 2025-11-06

**Authors:** Ivana Purnama Dewi, Andreas Mercyan Anggitama, Teuku Yusrizal, Kristin Purnama Dewi

**Affiliations:** a Faculty of Medicine, Duta Wacana Christian University, Yogyakarta, Indonesia; b Department of Cardiology and Vascular Medicine, Bethesda Hospital, Yogyakarta, Indonesia; c Department of Cardiology and Vascular Medicine, Dr Fauziah General Hospital, Bireuen, Indonesia; d Department of Pulmonology and Respiratory Medicine, Bethesda Hospital, Yogyakarta, Indonesia

**Keywords:** cardiovascular, electric vehicles, electromagnetic field

## Abstract

Electric vehicles (EVs) are increasingly adopted worldwide as a sustainable alternative to internal combustion engine vehicles. Alongside their environmental advantages, concerns have emerged regarding the potential health impacts of electromagnetic field (EMF) exposure generated by EV components and charging processes. This review aims to summarize current evidence on cardiovascular effects associated with EMF exposure from EVs. Relevant studies from PubMed, ScienceDirect, and SCOPUS were evaluated for EMF exposure levels, cardiovascular outcomes, and potential interference with medical devices. Most studies confirm that EMF exposure from EVs remains within international safety standards. Some findings suggest possible biological effects, including oxidative stress and subtle electrocardiogram alterations, though no significant electromagnetic interference has been observed in patients with cardiac implants. Overall, no immediate cardiovascular risks have been identified. Nonetheless, the long-term effects of EV-related EMF exposure remain uncertain, highlighting the need for further longitudinal studies to assess chronic exposure and its potential consequences to support the development of comprehensive safety guidelines.

Electric vehicles (EVs) are rapidly transforming the transportation sector and are expected to dominate global markets within the next decade. Their adoption is largely driven by the urgent need to mitigate climate change and reduce global urban air pollution.^1^ Several studies estimate that EVs can reduce carbon emissions by 23%-45% over their lifecycle compared to internal combustion engine vehicles, making them an important solution to global sustainability challenges.^2^^,^^3^

Beyond their benefits in reducing tailpipe emissions, EVs are not entirely free from environmental health concerns. Evidence indicates that they continue to produce nonexhaust emissions, such as particulate matter from tire and brake wear, resuspended road dust, and surface abrasion.^4^ These pollutants remain important contributors to the burden of cardiovascular disease. Compared with internal combustion engine vehicles, however, EVs are associated with lower levels of fine particulate matter, nitrogen oxides (NOx), and traffic noise—factors linked to reduced incidence of myocardial infarction, ischemic stroke, and noise-related hypertension.^5^, ^6^, ^7^ Electricity generation that still relies on fossil fuels can partially offset these benefits by releasing NOx, sulfur dioxide (SO_2_), and secondary particulates, which promote endothelial dysfunction, systemic inflammation, and oxidative stress.^6^^,^^8^ These pathophysiological processes are well-established drivers of atherosclerosis, arrhythmias, hypertension, and heart failure progression.^6^^,^^8^ Thus, the health impacts of EVs should be considered within a broader framework that extends beyond tailpipe emissions and recognizes both the remaining risks of nonexhaust emissions and the substantial benefits of reduced air and noise pollution.

In addition to these environmental considerations, the rapid expansion of EVs use has raised questions regarding the health effects of electromagnetic fields (EMFs) generated by EV components. EVs generate EMFs from various components within their electrical systems, including battery packs, electric motors, inverters, and charging ports. These emissions usually occur in the low- to mid-frequency range and are continuously present during vehicle operation and charging.^9^^,^^10^ Several factors, such as vehicle design, battery capacity, motor placement, and the frequency of charging cycles, influence the intensity of EMF exposure.^10^ Furthermore, wireless charging technology, which relies on resonant inductive coupling, may contribute to increased EMF exposure within enclosed vehicle environments.^10^

Understanding the potential biological effects of EMFs requires a distinction between ionizing and nonionizing radiation.^11^ Whereas ionizing radiation is well known to exert harmful effects, the long-term consequences of nonionizing EMFs, such as those produced by EVs, remain uncertain. Intense and prolonged exposure to ionizing ions can profoundly affect tissues and disrupt electrical currents, thereby altering cellular function.^12^ As the prevalence of EVs and other EMF-emitting technologies increases, it becomes increasingly important to investigate and understand their potential impacts on human health and well-being.

The growing concern over EMF-related health effects has drawn particular attention to the cardiovascular system, because it is highly sensitive to electrical signals, because the heart functions through bioelectrical conduction regulated by ion channels and pacemaker cells.^13^ Disturbances from external EMF exposures may influence electrophysiological activity or interfere with cardiac implantable electronic devices (CIEDs) such as pacemakers and implantable cardioverter-defibrillators. This raises concerns for CIED recipients as a vulnerable group at risk due to their dependence on properly functioning medical devices.

Given the rapid growth of EVs and the limited but emerging research on their health implications, especially in cardiovascular systems, it is crucial to evaluate the current evidence systematically. This review aims to summarize available studies on EMF exposure from EVs, focusing on cardiovascular health and potential risks to patients with CIEDs, thereby providing insights relevant to clinicians, researchers, policymakers, and the general public.

## Methods

A systematic search was conducted to identify studies on EMF exposure from EVs and its health impacts. A literature search was conducted in PubMed, ScienceDirect, and SCOPUS using the following keywords: “electric vehicles” OR “electromagnetic radiation” AND “health impact” OR “cardiology” OR “cardiovascular” OR “implantable electronic device” OR “CIEDs” OR “pacemaker” OR “implantable cardioverter defibrillator” OR “ICD.”

Studies were selected based on predefined inclusion and exclusion criteria. The inclusion criteria were as follows: 1) studies in humans; 2) published within the last 10 years to ensure relevance to current EVs technology; 3) studies that quantify EMF exposure levels; 4) research that evaluates potential health impacts of EMF exposure from EVs; and 5) studies assessing medical device interference, particularly with implantable cardiac devices (eg, pacemaker, defibrillator). The exclusion criteria included: 1) studies that do not focus on EMF exposure from EVs; 2) research that only examines technical aspects of EVs (eg, motor efficiency, battery performance) without discussing health implications; and 3) research focusing on non-EVs emissions (eg, gasoline or diesel vehicles) without specific comparisons to EV-related exposures.

**Figure 1:**
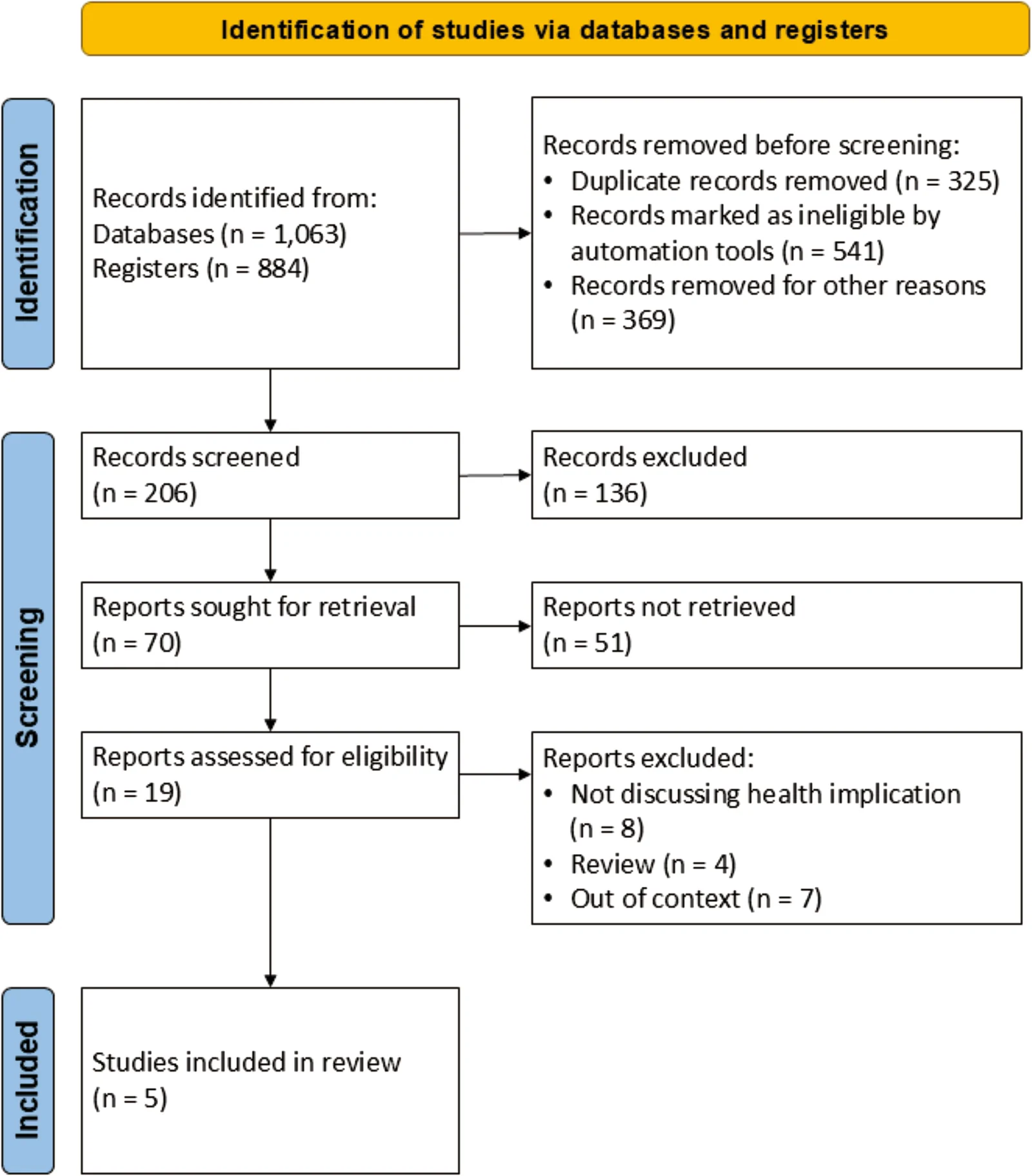
PRISMA Flowchart of Study Selection The PRISMA (Preferred Reporting Items for Systematic Reviews and Meta-Analyses) diagram summarizes the identification and selection of studies included in this review.

Studies were screened independently by 2 reviewers (A.M.A. and I.P.D.), and disagreements were resolved through discussion. Following the identification of eligible studies, data extraction was conducted to gather information on study design, sample population, exposure assessment, health outcomes, and key findings. A total of 1,947 records were identified, consisting of 1,063 from databases and 884 from registers. After removing 325 duplicate records and 541 records flagged as ineligible by automation tools, 369 additional records were excluded due to irrelevance, lack of full-text availability, or being conference abstracts without peer review. Despite the large initial data set, strict inclusion criteria ensured that only high-quality, relevant primary studies were considered. The PRISMA (Preferred Reporting Items for Systematic Reviews and Meta-Analyses) flowchart (Figure 1) visually represents the study selection process.

## Results

The collected studies comprehensively examine the EMF exposure and its potential cardiovascular impacts, covering various aspects, including exposure levels, health risks, and medical device interference. Findings from the selected studies are summarized in Table 1.^14^, ^15^, ^16^, ^17^, ^18^

**Table 1:** Summary of Various Studies Included in the Systematic Review

First Author, Year	Study Design	Sample Population	Exposure Assessment	Health Outcome	Key Findings
Fang et al, 2016^14^	Single blind experimental study	22 healthy volunteers	ELF-PEMF exposure at different intensities	Changes in ECG time intervals and RMS values	• The RMS values of the ECG signals increased by an average of 3.72% after short-term ELF-PEMF exposure for the majority (81.8%) of participants • The only ECG interval affected by the ELF-PEMF exposure was small change of RR interval, whereas other intervals, such as the PR, RT, and QT intervals, were not significantly affected
Napp et al, 2014^15^	Clinical in vivo provocation study	110 patients with pacemaker/ICD	Extremely low-frequency EMFs	Potential atrial/ventricular interference in strong EMFs	• Extremely low-frequency EMFs in everyday life do not disturb ICD sensing capabilities • Strong EMFs in occupational environments can cause inappropriate sensing and false detection of arrhythmias • The atrial lead is more susceptible to EMI than the ventricular lead
Lennerz et al, 2018^16^	Cross-sectional evaluation	108 patients with CIEDs	Exposure to EMFs inside EVs (BMW i3, Nissan Leaf, Tesla Model 85S, VW e-up!) during operation and charging	Detection of EMI with CIEDs	• No EMI was detected in any patient (0% incidence; 95% CI: 0%-3.4%) • Highest EMF exposure occurred during charging is 30.1-116.5 μT, but did not affect device function • No pacing inhibition, inappropriate shocks, or device reprogramming observed • EVs shielding likely protects against EMI, making them safe for CIED patients
Lennerz et al, 2023^17^	Observational study	130 patients with CIEDs performing 561 charging events	High-power EVs charging (up to 350 kW) and potential EMI on CIEDs	Detection of EMI effects on pacemakers or defibrillators	• There was no evidence of EMI in patients with CIEDs during high-power charging of EVs • The risk of EMI was very low, with a 95% CI of 0%-2% on a patient-based analysis and 0%-0.6% on a charge-based analysis
Wase et al, 2023^18^	Proof-of-concept study	69 patients with ICD	Exposure to EMI during the charging of a Tesla EV (Model S and X) in various positions in and around the car	Detection of EMI effects on ICD function	• No EMI effects were observed on ICD function at either nominal or highest sensitivity settings • No inappropriate shocks, no damage to ICD, and no interference were detected at any of the 5 tested positions around the vehicle

CIED = cardiac implantable electronic device; ECG = electrocardiogram; ELF-PEMF = extremely low-frequency pulsed electromagnetic field; EMF = electromagnetic field; EMI = electromagnetic interference; EV = electric vehicle; ICD = implantable cardioverter-defibrillator; RMS = root mean square.

## Discussions

### EMF exposure characteristics in EVs

EVs are widely regarded as a cleaner alternative; however, uncertainties remain regarding the long-term effects of chronic EMF exposure, particularly its impact on cardiovascular health and medical devices. Whereas the research on general cardiovascular effects of EMFs generated by EVs remains limited, some studies suggest potential health implications that warrant further investigation.^19^

EMFs generally are classified into static magnetic fields (0 Hz), extremely low-frequency EMFs (1–300 Hz), intermediate-frequency fields (300 Hz-10 MHz), and radiofrequency fields (10 MHz-300 GHz).^20^ EMFs produced by EV components fall primarily within the extremely low- to intermediate-frequency ranges.^21^ These field are nonionizing and nonthermal, so they do not directly damage DNA but may influence biological processes through subtle electrophysiological and biochemical mechanisms.^21^ Previous studies have examined these frequency ranges for potential biological effects, with some evidence suggesting that long-term exposure, even at low intensities, could alter cardiovascular parameters.^19^^,^^22^

Studies have shown that EMF exposure is highest near the inverter and electric motor, particularly in the driver and passenger seats.^12^ Although, field strength decreases with distance, but the absorption of electromagnetic energy varies in tissues and organs due to differences in their dielectric properties.^22^ Despite these differences, measured EMF levels remain below International Commission on Non-Ionizing Radiation Protection safety standards for public exposure.^12^^,^^23^ When comparing vehicle types, EVs generally emit higher low-frequency EMFs than internal combustion engine vehicles.^10^^,^^24^ Meanwhile, measurements in hybrid EVs indicate similar or slightly lower EMF strengths compared to EVs.^25^ This finding suggests that electromagnetic exposure is influenced more by powertrain design than solely by the presence of an electric motor.^25^

### Biological mechanisms and vascular implications of EMF from EVs

Several mechanisms have been proposed to explain how EMFs may affect cardiovascular health (Central Illustration). Chronic exposure to extremely low-frequency EMFs can impair endothelial function by altering membrane electrophysiology and disrupting nitric oxide (NO) production.^26^^,^^27^ These fields induce a frequency- and amplitude-dependent depolarization of endothelial cell membranes, which could alter calcium ion channels in myocardial cells that play a crucial role in cardiac excitability and contraction.^28^^,^^29^ Dysregulation of calcium signaling may disrupt electrical activity of cardiac cells, potentially increasing susceptibility to arrhythmias, such as atrial fibrillation, ventricular arrhythmias, or conduction abnormalities.^29^

**Central Illustration:**
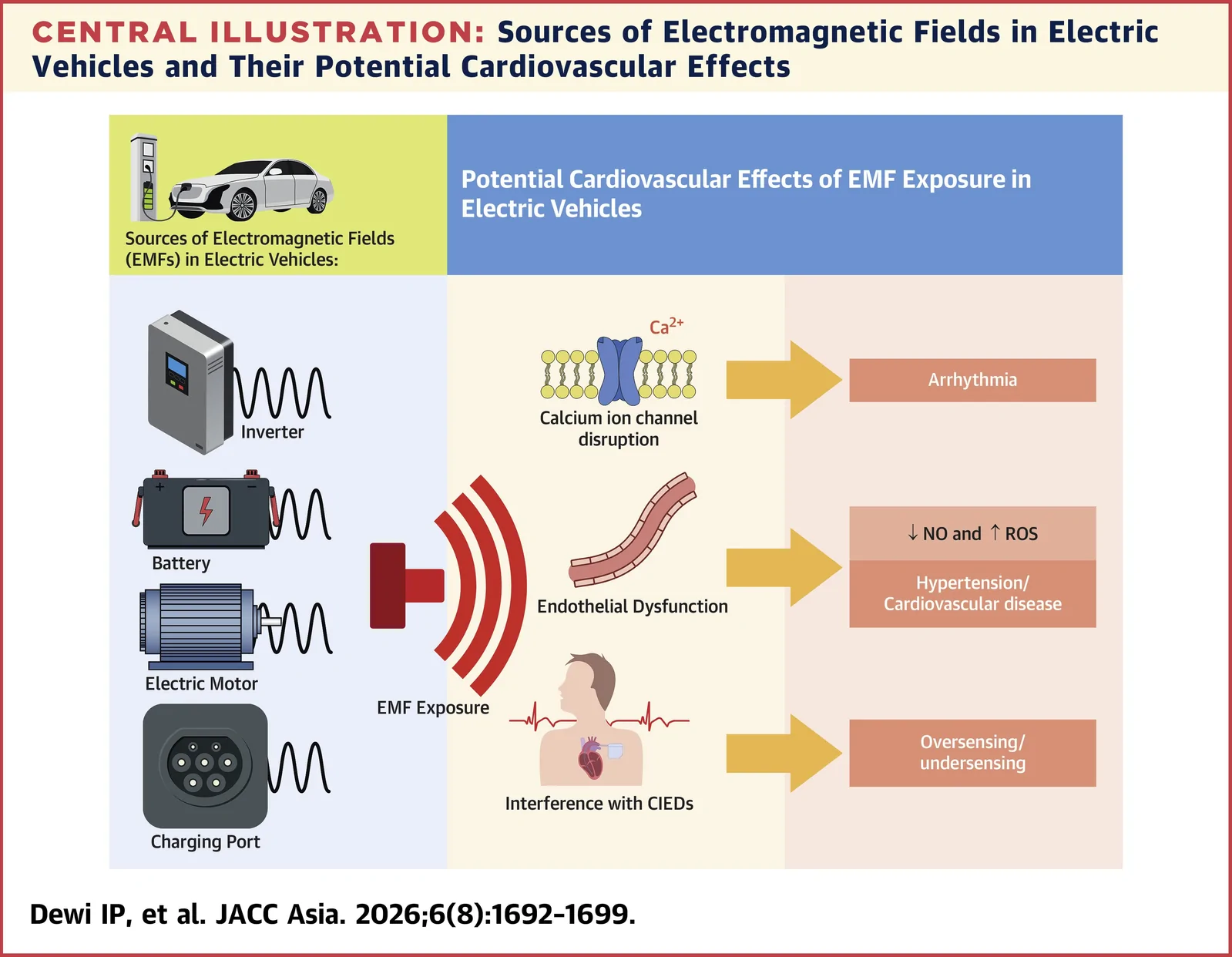
Sources of Electromagnetic Fields in Electric Vehicles and Their Potential Cardiovascular Effects Electromagnetic field (EMF) exposure from electric vehicles (EVs) primarily originates from key components such as the inverter, battery, electric motor, and charging port. EMFs may affect cardiovascular health through 3 main mechanisms: 1) calcium channel disruption, which may lead to arrhythmias; 2) endothelial dysfunction, characterized by reduced NO and increased reactive oxygen species (ROS), potentially contributing to hypertension and cardiovascular disease; and 3) interference with cardiac implantable electronic devices (CIEDs), leading to possible undersensing or oversensing. Further research is needed to evaluate the long-term cardiovascular implications of chronic EMF exposure from EVs.

Calcium influx is also essential for activating endothelial NO synthase, therefore, disruption of this process can reduce NO production and contribute to increased vascular stiffness.^25^^,^^26^ An experimental study in rats demonstrated that prolonged exposure to extremely low-frequency EMFs increased reactive oxygen species generation, which enhances oxidative stress and facilitates superoxide-mediated degradation of NO.^30^ A study by Morimoto et al,^31^ found that EMF exposure can impair endothelial function by modulating the production of endothelin-1, a potent vasoconstrictor, thereby shifting the vascular balance toward impaired vasodilation and dysfunction. In that study, EMFs significantly suppressed thrombin-induced endothelin-1 production and messenger RNA expression in various endothelial cell types.^31^ However, this effect of EMFs was abolished when NO synthase was inhibited, indicating that this mechanism is NO dependent.^31^ These findings suggest that EMFs not only alter endothelin-1 signaling but also alter NO signaling. Disruption of NO signaling reduces NO availability, leading to impaired vasodilation and contributing to increased vascular stiffness, highlighting the potential role of extremely low-frequency EMF exposure in the development of vascular dysfunction and hypertension.^26^^,^^31^ Collectively, these studies highlight that chronic EMF exposure may impair vascular homeostasis by disrupting the balance between vasodilatory and vasoconstrictive factors.

Although current clinical studies reported that EMF exposure in EVs do not have any harmful cardiovascular effects, the long-term implications remain uncertain. Chronic, daily exposure could potentially convert these biological alterations into clinically relevant outcomes, particularly as EV adoption continues to grow worldwide. Longitudinal studies with prolonged follow-up are urgently necessary to ascertain whether chronic EMF exposure contributes to subtle but important cardiovascular outcomes such as ischemic events, arrhythmias, or hypertension.

### Interference with cardiac devices

EMFs may have a potential risk of disrupting cardiac electrical activity, particularly in individuals with CIEDs who may be especially vulnerable to EMF interference. A systematic review of the literature indicates that electromagnetic interference (EMI) may be particularly provoked by certain components and systems, including security systems and induction technologies that are found in EVs.^32^ The likelihood of EMI depends on several factors, including exposure-related parameters (frequency, field strength, and modulation) and implant-related parameters (type of implant, model, lead configuration, implantation site, and sensitivity settings).^32^

EMI with CIEDs can occur through various mechanisms that disrupt their normal function, potentially leading to inappropriate pacing, device malfunction, or even life-threatening arrhythmias.^33^ The primary mechanism underlying EMI in CIEDs is the induction of electrical currents within the leads.^32^ Strong EMF sources, particularly in occupational and environmental settings, have been shown to cause oversensing or undersensing in CIEDs.^15^ In such environments, the leads may act as antennas, transmitting unintended signals to the device’s sensor circuitry. As a result, the device may misinterpret these signals as intrinsic cardiac activity, potentially leading to inappropriate responses, such as pacing inhibition or the unnecessary delivery of shocks.^32^

Recent studies reported that low-frequency EMFs (<1 kHz) can interfere with CIEDs.^32^^,^^34^ Wireless power transmission systems used in EVs also can induce localized heating around metallic implants, with the potential to exceed safety limits in certain scenarios.^35^ However, a study by Lennerz et al,^17^ found no significant EMI in patients with pacemakers and defibrillators using high-power EVs chargers, with no occurrences of pacing inhibition, inappropriate tachycardia detection, or spontaneous reprogramming. Another in vivo study evaluating the impact of 50-Hz EMF exposure on implantable cardioverter-defibrillators found that whereas nominal sensitivity settings provided significant protection, maximum sensitivity settings increased the likelihood of atrial lead interference.^15^ Given the increasing adoption of EVs and their associated charging infrastructures, further research is needed to assess the safety thresholds of EMF exposure for individuals with cardiac devices, particularly in environments with high EMF emissions, such as those associated with EV charging stations. However, evidence from individual case reports remains scarce, suggesting the need for future case-based documentation to provide broader clinical insights, especially for patients with CIEDs.

### Regulatory standards and safety considerations

To mitigate the risks associated with EMFs, the International Commission on Non-Ionizing Radiation Protection has established guidelines and standards to limit human exposure.^23^ Although these guidelines recommended exposure levels of EMFs, including those generated by EVs, there are no definite limits concerning cardiovascular consequences and specifically addressing CIEDs. Most of these recommendations are derived from data in the general population, whereas clinical evidence in vulnerable groups, such as individuals with cardiovascular disease or cardiac implants, remains limited. Susceptibility to EMFs can also vary depending on device type, implantation site, and individual comorbidities, making a 1-size-fits-all standard inadequate. This gap highlights the need of interdisciplinary collaboration between engineers, clinicians, and policy makers to ensure that evolving technologies remain compatible with patient safety. Future efforts should therefore focus not only on updating technical standards but also on conducting real-world monitoring to provide evidence-based protection for individuals with CIEDs. Moreover, the rapid development of EV technologies, including high-power and wireless charging systems, introduces new exposure scenarios that current guidelines may not fully address, further emphasizing the urgency for updated and adaptive safety regulations.

The evolution of CIED technology also reflects a growing awareness of EMF-related risks. The latest CIEDs are already equipped with advanced shielding mechanisms that enhance their resistance to EMF exposure and reduce the risk of EMI, thereby enhancing patient safety.^35^ Nevertheless, variations among CIED models and clinical settings indicate that susceptibility cannot be eliminated; therefore, individuals with cardiac implants are advised to consult their health care providers regarding the susceptibility of their specific device models to EMI. Clinicians should consider device programming strategies such as reducing sensitivity settings and prioritizing bipolar lead configurations. Additionally, patient education on maintaining safe distances from potential EMI is important, because the distance from high-field components is also a crucial factor in determining the risk of interference.^18^^,^^36^ Future research and policy development should focus on developing comprehensive safety standards to better minimize the impact of low-frequency EMFs on CIED function and overall patient safety.

## Conclusions

Given the rising adoption of EVs worldwide, regulators and manufacturers should collaborate to monitor EMF exposure levels and update safety guidelines accordingly. Whereas current evidence suggests that EMF exposure from EVs does not pose an immediate health risk, long-term effects remain uncertain. Given the increasing global adoption of EVs, future research should focus on longitudinal studies assessing chronic exposure to low-frequency EMFs. Public awareness campaigns could also help inform EV users about potential exposure risks, particularly for individuals with medical implants.

## Funding Support and Author Disclosures

The authors have reported that they have no relationships relevant to the contents of this article to disclose.

## References

[1] Zhi R., Luo W. (2022). 2022 14th International Conference on Computer Research and Development (ICCRD).

[2] Changzheng Z., Pengbo L. (2023). 2023 11th International Conference on Traffic and Logistic Engineering (ICTLE).

[3] Zhang X., Gao F., Gong X., Wang Z., Liu Y. (2018). Advances in Energy and Environmental Materials.

[4] Fussell J.C., Franklin M., Green D.C. (2022). A review of road traffic-derived non-exhaust particles: emissions, physicochemical characteristics, health risks, and mitigation measures. Environ Sci Technol.

[5] Woo S.H., Jang H., Lee S.B., Lee S. (2022). Comparison of total PM emissions emitted from electric and internal combustion engine vehicles: an experimental analysis. Sci Total Environ.

[6] Palacio L.C., Pachajoa D.C., Echeverri-Londoño C.A., Saiz J., Tobón C. (2023). Air pollution and cardiac diseases: a review of experimental studies. Dose Response.

[7] Tabaei S., Ghalenoo S.R., Panahandeh M., Bagheri G., Tabaee S.S. (2025). The relationship between noise pollution and cardiovascular diseases: an umbrella review on meta-analyses. BMC Cardiovasc Disord.

[8] Hamanaka R.B., Mutlu G.M. (2018). Particulate matter air pollution: effects on the cardiovascular system. Front Endocrinol (Lausanne).

[9] Yang L., Lu M., Lin J. (2019). Long-term monitoring of extremely low frequency magnetic fields in electric vehicles. Int J Environ Res Public Health.

[10] Dong X., Gao Y., Lu M. (2024). The electromagnetic exposure level of a pure electric vehicle inverter based on a real human body. Appl Sci.

[11] Tuieng R.J., Cartmell S.H., Kirwan C.C., Sherrat M.J. (2021). The effects of ionising and non-ionising electromagnetic radiation on extracellular matrix proteins. Cells.

[12] Gherardini L., Ciuti G., Tognarelli S., Cinti C. (2014). Searching for the perfect wave: the effect of radiofrequency electromagnetic fields on cells. Int J Mol Sci.

[13] Parizek D., Visnovcova N., Hamza Sladicekova K. (2023). Electromagnetic fields—do they pose a cardiovascular risk?. Physiol Res.

[14] Fang Q., Mahmoud S.S., Yan J., Li H. (2016). An investigation on the effect of extremely low frequency pulsed electromagnetic fields on human electrocardiograms (ECGs). Int J Environ Res Public Health.

[15] Napp A., Joosten S., Stunder D. (2014). Electromagnetic interference with implantable cardioverter-defibrillators at power frequency: an in vivo study. Circulation.

[16] Lennerz C., O'Connor M., Horlbeck L. (2018). Electric cars and electromagnetic interference with cardiac implantable electronic devices: a cross-sectional evaluation. Ann Intern Med.

[17] Lennerz C., Schaarschmidt C., Blažek P. (2023). High-power chargers for electric vehicles: are they safe for patients with pacemakers and defibrillators?. Europace.

[18] Wase A., Hussain U.A., Ratajczak T., Aung T.T., Ali O., Markert R.J. (2023). The impact of electromagnetic interference from charging all-electric vehicles on implantable cardioverter-defibrillator performance. J Innov Card Rhythm Manag.

[19] Elmas O. (2016). Effects of electromagnetic field exposure on the heart: a systematic review. Toxicol Ind Health.

[20] Modenese A., Gobba F. (2021). Occupational exposure to electromagnetic fields and health surveillance according to the European Directive 2013/35/EU. Int J Environ Res Public Health.

[21] Wang Y., Zhao Z.G., Chai Z., Fang J.C., Chen M. (2023). Electromagnetic field and cardiovascular diseases: a state-of-the-art review of diagnostic, therapeutic, and predictive values. FASEB J.

[22] Peyman A. (2011). Dielectric properties of tissues; variation with age and their relevance in exposure of children to electromagnetic fields; state of knowledge. Prog Biophys Mol Biol.

[23] International Commission on Non-Ionizing Radiation Protection (ICNIRP) (2010). Guidelines for limiting exposure to time-varying electric and magnetic fields (1 Hz to 100 kHz). Health Phys.

[24] Gryz K., Karpowicz J., Zradziński P. (2022). Complex electromagnetic issues associated with the use of electric vehicles in urban transportation. Sensors (Basel).

[25] Pääkkönen R., Korpinen L. (2019). Low frequency magnetic fields inside cars. Radiat Prot Dosimetry.

[26] Trivedi D., Hallock K., Bergethon P. (2013). Electric fields caused by blood flow modulate vascular endothelial electrophysiology and nitric oxide production. Bioelectromagnetics.

[27] Azab A.E., Ebrahim S.A. (2017). Exposure to electromagnetic fields induces oxidative stress and pathophysiological changes in the cardiovascular system. J Appl Biotechnol Bioeng.

[28] Pall M.L. (2013). Electromagnetic fields act via activation of voltage-gated calcium channels to produce beneficial or adverse effects. J Cell Mol Med.

[29] Landstrom A.P., Dobrev D., Wehrens X.H.T. (2017). Calcium signaling and cardiac arrhythmias. Circ Res.

[30] Akdag M.Z., Bilgin M.H., Dasdag S., Tumer C. (2007). Alteration of nitric oxide production in rats exposed to a prolonged, extremely low-frequency magnetic field. Electromagn Biol Med.

[31] Morimoto S., Takahashi T., Shimizu K. (2005). Electromagnetic fields inhibit endothelin-1 production stimulated by thrombin in endothelial cells. J Int Med Res.

[32] Driessen S., Napp A., Schmiedchen K., Kraus T., Stunder D. (2019). Electromagnetic interference in cardiac electronic implants caused by novel electrical appliances emitting electromagnetic fields in the intermediate frequency range: a systematic review. Europace.

[33] Napp A., Stunder D., Maytin M., Kraus T., Marx N., Driessen S. (2015). Are patients with cardiac implants protected against electromagnetic interference in daily life and occupational environment?. Eur Heart J.

[34] Fukuta M., Mizutani N., Waseda K. (2005). Influence of electromagnetic interference on implanted cardiac arrhythmia devices in and around a magnetically levitated linear motor car. J Artif Organs.

[35] Shah I.A., Yoo H. (2020). Assessing human exposure with medical implants to electromagnetic fields from a wireless power transmission system in an electric vehicle. IEEE Trans Electromagn Compat.

[36] Ramya K., Gopalakrishnan J., Chokkalingam B., Verma R., Mihet-Popa L. (2025). A comprehensive evaluation and assessment practices of electromagnetic interferences in electric vehicle. IEEE Access.

